# Deciphering the role of receptor-like kinases in the adaptation of *Orinus* species to the habitat of the Qinghai-Xizang (Tibet) Plateau

**DOI:** 10.3389/fpls.2026.1810781

**Published:** 2026-04-29

**Authors:** Yue Fang, Qinyue Min, Kaifeng Zheng, Yanrong Pang, Yanfen Zhang, Feng Qiao, Jinyuan Chen, Yuping Liu, Xu Su, Shengcheng Han

**Affiliations:** 1 Key Laboratory of Biodiversity Formation Mechanism and Comprehensive Utilization of the Qinghai-Tibet Plateau in Qinghai Province, College of Life Sciences, Qinghai Normal University, Xining, Qinghai, China; 2 Beijing Key Laboratory of Gene Resources and Molecular Development, College of Life Sciences, Beijing Normal University, Beijing, China; 3 Academy of Plateau Science and Sustainability of the People’s Government of Qinghai Province and Beijing Normal University, Qinghai Normal University, Xining, Qinghai, China

**Keywords:** DEGs (differentially expressed genes), Orinus, Qinghai-Xizang (Tibet) Plateau, receptor-like kinase, speciation adaptation

## Abstract

Receptor-like kinases (RLKs) play vital roles in development, stress perception and signal transduction, acting as core components in abiotic stress responses in plants. As important regulators of plant adaptation to harsh environments, RLKs are particularly critical for extremophytes surviving extreme cold, drought, and high-UV stress. However, the RLK gene family in extremophytes, such as Orinus species distributed in Qinghai-Xizang (Tibet) Plateau (QTP), remains largely uncharacterized. Given the unique adaptation of Orinus to the extreme QTP habitat, investigating the RLK gene family is essential to uncover the molecular basis of its stress tolerance and high-altitude adaptation. Here, we performed a comprehensive genome-wide analysis of RLK genes in Orinus (ORLKs) to identify a total of 647 ORLK candidates classified into 52 subfamilies. Phylogenetic and synteny analyses revealed a close evolutionary relationship between Orinus and wheat (Triticum aestivum), with extensive collinearity detected among five core subfamilies. Moreover, the ORLKs exhibited an uneven chromosomal distribution, accompanied by tandem duplication events. To assess the potential function of these ORLKs, we compared the expression profiles between two species of Orinus (O. thoroldii and O. kokonoricus) and identified 120 differentially expressed ORLK genes (DEGs). Cis-acting element analysis of DEG promoters revealed a significant enrichment of hormone-responsive, stress-responsive and light-responsive elements, indicating the complexity of their regulatory networks. This study provides insights into the molecular mechanisms of adaptation to extreme environments in Orinus on QTP, revealing evolutionary innovations in RLKs that could inform strategies for engineering stress-tolerant crops, and our findings establish a functional genomic foundation for RLK-mediated signaling in extremophytes.

## 1 Introduction

The growth and development of plants always face the dual challenges of both abiotic and biotic stresses, such as environmental pressures like drought, low temperature, and salinity, as well as biological threats including insect pests and various microbial infections (Gong et al., 2020). These adversities not only impair plant growth, yield, and quality but also threaten global ecological stability (Jindo et al., 2023). Unlike mammals, plants lack a circulating immune system; instead, they rely on cell-surface Receptor-like kinases (RLKs)—functional analogs of animal receptor tyrosine kinases (RTKs) to initiate immune and stress-responsive signaling cascades (Ding et al., 2024). RLKs constitute the largest family of receptor proteins in plants and play a central role in sensing and responding to environmental cues (Gandhi and Oelmüller, 2023). Structurally, RLKs are transmembrane proteins. They typically possess an extracellular domain for ligand binding, a single transmembrane helix, and an intracellular kinase domain that enables them to directly connect external stimuli to internal cellular signaling cascades (Jose et al., 2020). RLKs play an important role in detecting developmental signals, regulating plant growth and reproduction, dictating stomatal patterning, guiding pollen tube growth, facilitating symbiosis, and mediating hormone signaling. Additionally, they are pivotal in enabling plants to adapt to various biotic and abiotic stresses, ensuring the organism’s survival and success in diverse environmental conditions (Wan et al., 2008; Breiden and Simon, 2016).

Research on RLKs is well-established in key model plants and several other species. Over 600 members identified In *Arabidopsis thaliana* (Zhao et al., 2025). In 2018, Lin et al. identified 563 RLK genes in Jilin ginseng (*Panax ginseng* C.A. Meyer) (Lin et al., 2018). RLKs are categorized into multiple subfamilies based on their extracellular domains including Protein kinase R-like endoplasmic reticulum kinase (PERK), Extensin, and Catharanthus roseus receptor-like kinase 1-like (CrRLK1L), each exhibiting distinct ligand specificities and biological functions (Vaattovaara et al., 2019). In drought stress responses, RLKs play well-documented roles. For example, *Arabidopsis* LRR-RLK GHR1 modulates drought-induced stomatal closure via hydrogen peroxide signaling (Takada et al., 2007), Cysteine-rich receptor-like kinase (CRKs) regulate ROS homeostasis and cell death under water deficit (Hsu et al., 2021; Kimura et al., 2020) and soybean LecRLK SD1–29 enhances drought tolerance by altering ABA sensitivity (Saleem et al., 2022). Despite these advances, most RLK studies have focused on model species or crops, creating a critical knowledge gap in RLK evolution and function in extremophytes like *Orinus*.

*Orinus*, commonly known as sand grass, is a perennial herb within the grass family (Poaceae) characterized by its elongated rhizomes, flat or involute leaf blades, and a paniculate inflorescences bearing narrowly oblong caryopses. It is well-adapted to warm, arid sandy soils and typically inhabits dry steppe environments at elevations ranging from 3,650 to 4,300 meters, and widely distributed across the Qinghai, Sichuan, Gansu, and Xizang regions (Liu et al., 2019). *Orinus* comprises three recognized species: *O. kokonoricus*, *O. intermedius*, and *O. thoroldii*. *Orinus* plays a vital role in erosion control due to its strong soil-binding capacity and high trampling tolerance. It effectively mitigates both water loss and wind-driven soil erosion in fragile arid landscapes (Su et al., 2022). This genus also demonstrates robust drought resistance and thrives in nutrient-poor substrates, making it valuable for desertification prevention. Furthermore, *Orinus* contributes to soil improvement through growth and litter input, enhancing soil organic matter, structure, water retention, and fertility (Qu et al., 2023). It provides habitat for various fauna, supporting local biodiversity and ecosystem stability. With its capacity for both sexual and asexual reproduction, *Orinus* frequently forms characteristic temperate shrub-steppe communities in association with species like *Sophora moorcroftiana*. These ecological traits, along with its value as high-quality plateau forage, establish *Orinus* as an important model for studying plant adaptation to extreme habitats on QTP (Su et al., 2015).

In order to explore the potential roles of RLKs in *Orinus* adaptation on QTP, we herein conducted a genome-wide identification of RLK genes in *Orinus*, yielding 647 *Orinus* RLK (ORLK) candidate genes. These ORLKs were then systematically analyzed across multiple dimensions, including their physicochemical properties, predicted subcellular localization, and phylogenetic relationships. We further examined conserved motifs, gene structures, and kinase domain features. In addition, interspecific collinearity analysis was conducted to explore ORLK gene conservation between *Orinus* and related species. We also assessed their expression patterns using available transcriptomic data to characterize differential expression under relevant conditions. This study lays a foundation for elucidating the evolutionary adaptation and stress-resistant functions of ORLK genes, and further deepens the understanding of the molecular mechanisms underlying the adaptation of desert plants to extreme habitats.

## 2 Materials and methods

### 2.1 Whole genome identification of ORLKs in *Orinus*

Protein sequences of *Orinus* were downloaded from the National Genomics Data Centre under accession PRJCA018722 (Qu et al., 2023). Using BLASTP (v2.16.0) to conduct searches against a subject database of *Arabidopsis*, rice, and wheat RLK protein sequences to enhance detection sensitivity (Chen et al., 2022). All candidate were validated through conserved domain analysis using InterProScan (v5.75−106.0), confirming their structural integrity as functional ORLKs (Jones et al., 2014).

### 2.2 Characteristic information of ORLK families

The isoelectric point, molecular weight, and instability analyses of ORLKs were performed using the online tool ExPASy (https://web.expasy.org/protparam/). The subcellular localization of the ORLK genes were predicted via the WoLF PSORT website (https://wolfpsort.hgc.jp/).

### 2.3 Phylogenetic analysis and classification of ORLK families

We generated multiple sequence alignments of ORLKs using Multiple Alignment using Fast Fourier Transform (MAFFT: https://www.ebi.ac.uk/Tools/msa/mafft/) (v7.511) with the FFT-NS-2 strategy (Katoh and Standley, 2013). The resulting alignments were used to construct maximum-likelihood phylogenetic trees with IQ-TREE (v1.6.12) in TBtools-II based on 1000 bootstrap replicates (Chen et al., 2022). The G model was employed to account for rate heterogeneity across sites, and the JTT substitution matrix was used for amino acid replacements (Zoller and Schneider, 2012). Phylogenetic trees were visualized and annotated using interact Tree Of Life (iTOL: v7.51) for comprehensive comparative analysis (Zhou et al., 2023).

### 2.4 Gene and proteins structure analysis

To identify conserved motifs in ORLK proteins, the Multiple Expectation Maximization for Motif Elicitation (MEME) suite (v5.2.0) was employed with the following parameters: optimal motif width ranging from 10 to 50 amino acids, maximum number of motifs set to 10 and other configurations remained default (Bailey et al., 2009). Functional domains were predicted and annotated using the Conserved Domain Database (CDD) in NCBI. A schematic diagram of the protein structure was generated using TBtools-II.

### 2.5 Analyses of synteny in *Orinus*, wheat and *Arabidopsis*

Information on the physical locations of five ORLKs subfamily genes were retrieved from the *Orinus* genome database. To investigate the homology of these genes among *Orinus*, wheat and *Arabidopsis*, the MCScanX program was employed with default parameters. Syntenic relationships between the three species were visualized using TBtools-II.

### 2.6 Nonsynonymous (Ka)/Synonymous (Ks) analysis of ORLKs

Ka and Ks substitution rates were calculated for 1:1 orthologous ORLK gene pairs between *Orinus* and wheat using TBtools. Briefly, orthologs were identified with OrthoFinder2, and the corresponding CDS sequences were extracted. The Ka/Ks ratios were estimated using the YN model implemented in TBtools. After filtering by Ks < 5 and Ka/Ks < 2, the results were visualized as a density plot in R.

### 2.7 Expression patterns of DEGs

The expression patterns of all the ORLK families in the two *Orinus* species at different altitudes were investigated using public RNA-Seq data. Significant DERLKs in the two *Orinus* species were determined based on FPKM values. Our screening criteria were standard | log2 (fold change) | values ≥ 1 and p-values < 0. 05 (Zhao et al., 2025).

### 2.8 Gene Ontology and Kyoto Encyclopedia of Genes and Genomes analysis of DEGs

Functional annotation of DEGs were performed using eggNOG-mapper v2.1.13 against the eggNOG database (Huerta-Cepas et al., 2019). GO and KEGG enrichment analyses were then conducted using the TBtools.

### 2.9 *Cis*-elements analysis

The transcription start site (TSS) was determined based on the gene structure annotation of the Orinus genome. For each gene, the 5′-end of the annotated mRNA or CDS sequence was defined as the TSS, and the 2000 bp upstream region from this site was extracted as the promoter sequence for cis-element analysis. The promoter sequences of ORLK genes, which measure 2.0 kb in length and are situated upstream of the TSS, were extracted from the genome sequence using TBtools, based on the positional records present in the genome annotation file and submitted to the PlantCARE database (http://bioinformatics.psb.ugent.be/webtools/plantcare/html) for the prediction of promoter cis-acting elements.

## 3 Results

### 3.1 Identification, classification, and evolutionary analysis of ORLKs

**Figure 1:**
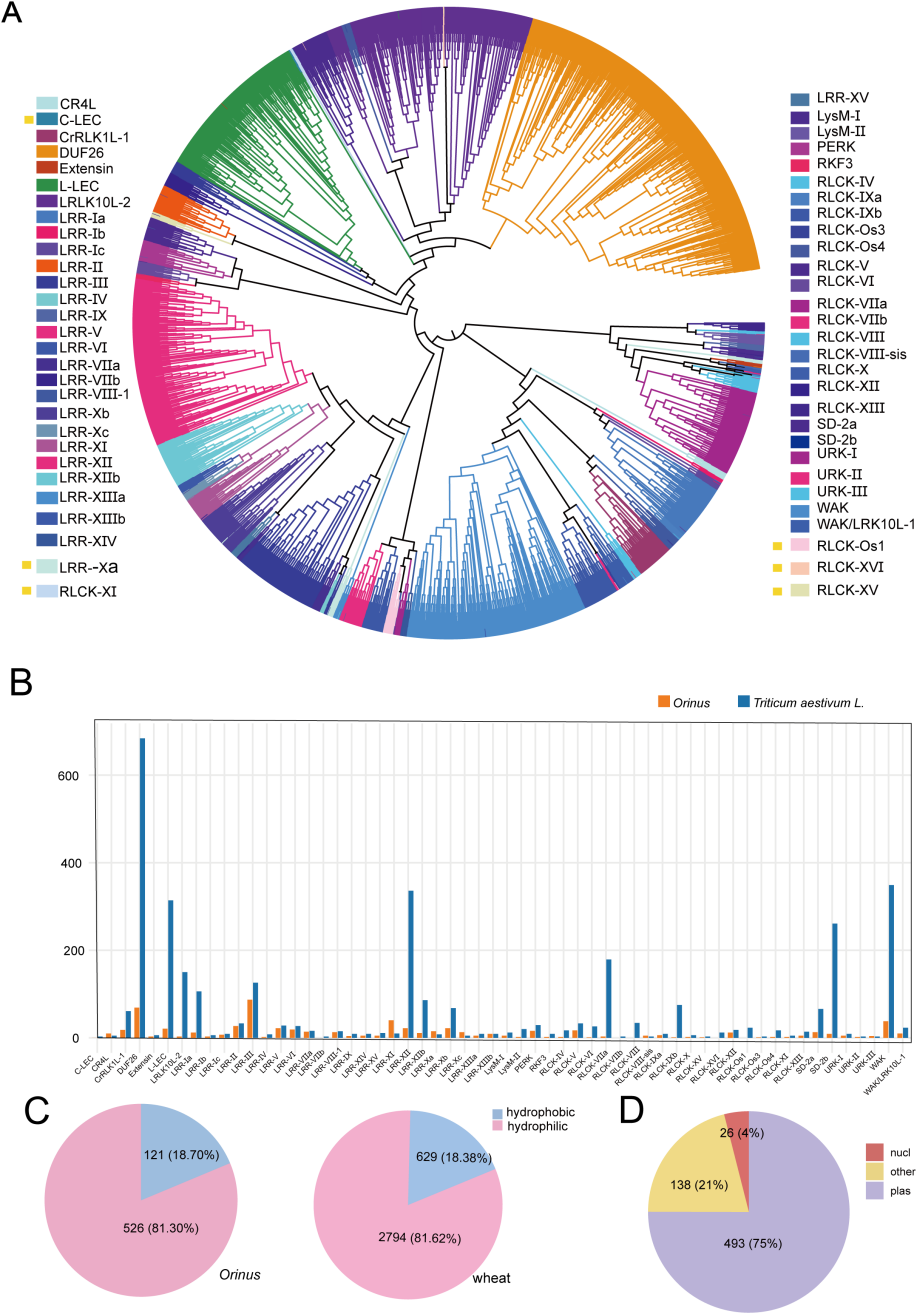
Classification of ORLK subfamilies. **(A)** Phylogenetic tree and subfamily classification of RLK in *Orinus* and wheat (bootstrap=1000). **(B)** The number of RLK in *Orinus* and wheat. **(C)** Proportion of average hydrophobicity and hydrophilicity of RLK in *Orinus* and wheat. **(D)** Pie chart of subcellular localization prediction for ORLK.

A total of 647 ORLK candidates were identified via a dual-approach strategy for gene identification. The homology search utilized a custom reference database containing 5,526 know RLK sequences that came from three model species — 610 from *Arabidopsis*, 3,424 from *Triticum aestivum* (wheat), and 1,492 from *Oryza sativa* (rice). Then, all initial candidates were further screened using the HMMER tool to screen two essential kinase domains (PF00069 and PF07714) (**Supplementary Table 1**) (El-Gebali et al., 2019; Hanada et al., 2008). The RLK complement of *Orinus* was defined by 52 subfamilies, constituting a fully shared subset of the 58 present in wheat (Zhao et al., 2025) and lacking six wheat-specific lineages (C-LEC, LRR-Xa, RLCK-XI, RLCK-XV, RLCK-XVI and RLCK-Os1) (**Figure 1A**). The absence of the RLK families in *Orinus* likely reflects they originated after the evolutionary divergence between wheat and *Orinus* within the monocot lineage. We then quantified the size distribution of RLK subfamilies in the *Orinus* genome. The total number of ORLK genes was approximately one-fifth that of wheat. The largest subfamily, LRR-III, contained 89 genes. This is followed by the DUF26 subfamily with 69 genes (**Figure 1B**). Moreover, we characterized the protein features of the RLK members. The ORLK proteins ranged from 210 to 3,483 amino acids in length, with the molecular weights between 22.3 kDa and 383.5 kDa and isoelectric points varied from 4.8 to 12.1 (**Supplementary Table 2**). The majority of ORLKs exhibited negative average hydropathicity values, accounting for 81.30% of the 526 ORLKs, which is similar with 81.62% of the 2,794 wheat RLKs. The remaining minority in each species were hydrophobic (**Figure 1C**). Localization screening identified 75% (493/647) ORLKs as plasma membrane-localized, while 4% (26/647) were nuclear. The remaining 21% (138/647), localized to other cellular compartments (**Figure 1D**, **Supplementary Table 3**). These results establish that the adaptive evolution of *Orinus* is driven by distinct genomic evolutionary strategies and the optimization of key RLK characteristics. To understand how gene family expansion correlates with functional adaptation, we selected several ORLK subfamilies, LRR-III, LRR-XI, LRR-XII, DUF26, WAK, for in-depth study, as these subfamilies exhibited the most substantial expansion in the *Orinus* genome compared to other ORLK subfamilies.

### 3.2 Chromosome distribution and phylogenetic relationships of OLRR-III gene family

**Figure 2:**
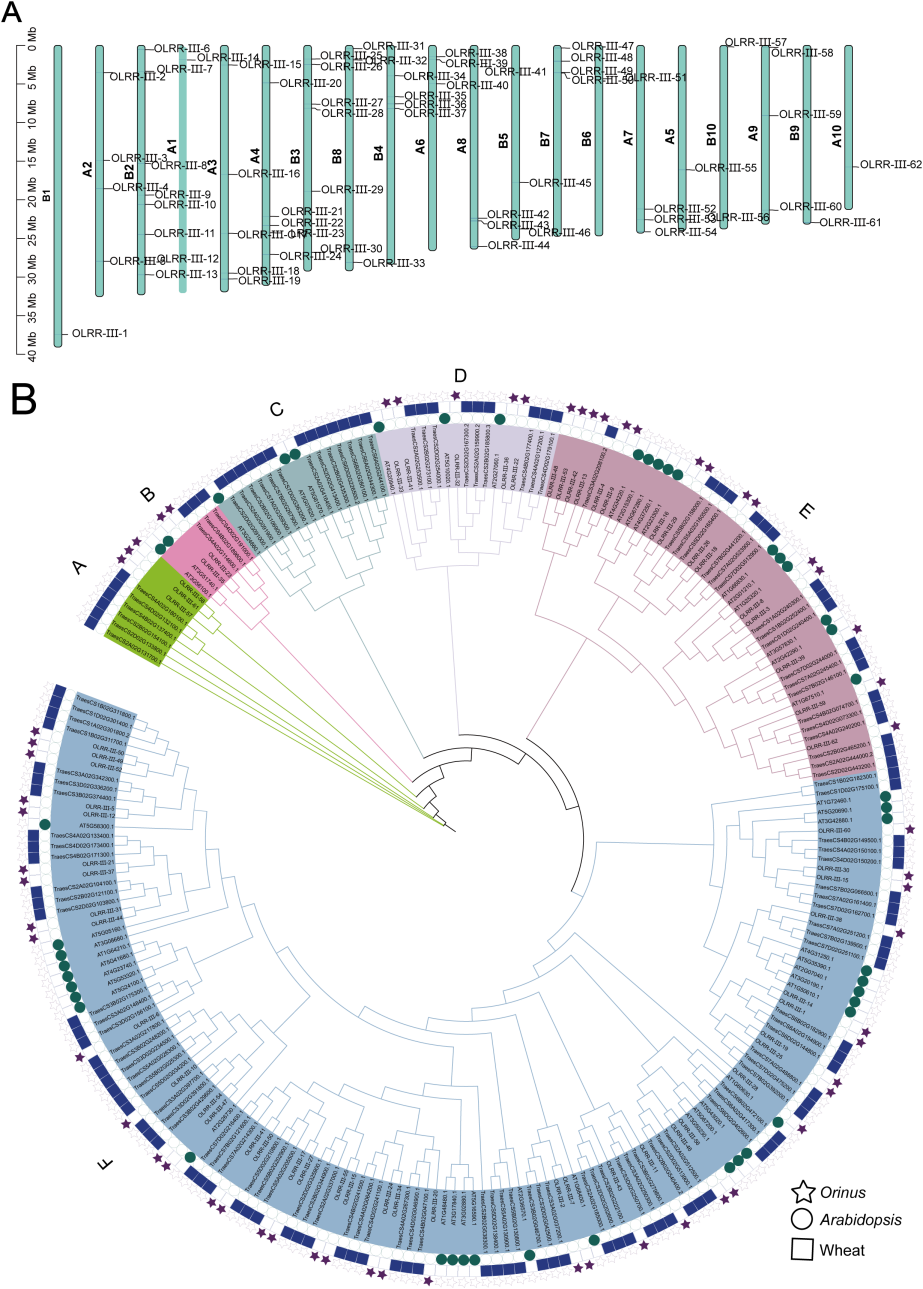
Evolutionary analysis of the LRR-III family. **(A)** Chromosomal distribution of OLRR-III genes. **(B)** Phylogenetic tree of LRR-III in *Orinus*, *Arabidopsis* and wheat. Stars represent *Orinus*, circles represent *Arabidopsis*, and squares represent wheat.

The 62 *Orinus* LRR-III (OLRR-III) genes, designated OLRR-III-1 to OLRR-III-62 based on chromosomal locations, were unevenly distributed across 20 chromosomes (**Figure 2A**). Major members of OLRR-III were found on B2 (8 genes) and B3 (6 genes), in stark contrast to the sparse representation (1 or 2 genes each) on eight other chromosomes, including A1, B1, A5, and B5. Only one tandem repeat gene pair was identified in the OLRR-III subfamily, namely OLRR-III-49 and OLRR-III-50. 12 homologous genes on five other chromosomes (OLRR-III-9/-10 on B2, OLRR-III-18/-19 on A3, OLRR-III-25/-26 on B3, OLRR-III-27/-28 on B3, OLRR-III-38/-39 on A6, OLRR-III-42/-43 on A8) were also detected. The biased chromosomal distribution of OLRR-III subfamily implied potential functional differentiation of these genes in different genomic regions.

To clarify the evolutionary relationships within the LRR-III gene family, we carried out a systematic phylogenetic analysis, incorporating not only the OLRR-III genes we identified but also homologous sequences from wheat and *Arabidopsis*. The maximum-likelihood phylogenetic tree showed that the OLRR-III gene family was divided into 6 subfamilies (Named Group A–F), with clear evolutionary divergence among the subfamilies and relatively high sequence conservation within each clade. Obviously, Group F represented the largest clade contained 38 *Orinus* genes and no homologous members were detected in *Arabidopsis*. A finding that points to gene family diversification following the monocot-dicot evolutionary split in the group A. The lack of *Orinus* genes in Group C implies that this gene loss event may be functionally permissible (**Figure 2B**).

### 3.3 The OLRR-XI family

The 40 OLRR-XI family genes exhibit an uneven distribution pattern across *Orinus* chromosomes. Genomic analysis revealed five tandem duplication events occurring in this family (**Supplementary Figure 1A**). This distribution pattern suggested that local gene duplication has actively contributed to the expansion of OLRR-XI. Comparative phylogenetic analysis with wheat and *Arabidopsis* revealed a striking, lineage-specific expansion of OLRR-XI in *Orinus* (**Supplementary Figure 1B**). Specifically, except for their complete absence from phylogenetic Group A, OLRR-XI genes far outnumber their orthologs in wheat and *Arabidopsis* across Groups B–E. Pronounced clade-specific expansion indicates that OLRR-XI genes evolved novel or enhanced functions to facilitate *Orinus* adaptation to extreme plateau environments.

In our investigation, we conducted a comprehensive analysis of the gene domains and the conserved motifs present within the OLRR-XI family. The types, numbers, and arrangement patterns of conserved motifs contained in different OLRR-XI genes showed significant differences. Meanwhile, Motif 1 and Motif 5 appeared at high frequency in most genes, emerged as the predominant motifs across genes (**Supplementary Figure 2**). In the domain analysis, the OLRR-XI family possessed the PLN00113 superfamily domain (**Supplementary Figure 3**).

### 3.4 The OLRR-XII family

Unlike the OLRR-XI family, the 22 OLRR-XII genes map to merely 10 chromosomes, which reflects a significant decrease in the chromosomal distribution range of this gene family (**Supplementary Figure 4A**). Despite overall reduction, the subfamily retains three tandem duplication events, with one notably forming an unusual four-gene OLRR-XII cluster. We speculate this cluster stems from recent localized expansion, which may confer selective advantages for fine-tuning specific stress responses. Phylogenetic analysis assigned the 22 OLRR-XII genes to four distinct groups (Group A-D) (**Supplementary Figure 2B**). Compared to wheat, *Orinus* and *Arabidopsis* exhibit marked OLRR-XII members reduction across these groups; this parallel contraction suggests a small core gene set suffices for basal functions, while wheat’s expansion reflects more complex immune/developmental needs.

The 10 conserved motifs of OLRR-XII genes vary in arrangement pattern and size across different genes. For instance, OLRR-XII-10 contains a greater number of motifs, with additional short motifs present at its terminal region; in contrast, the motif combination of OLRR-XII-7 is relatively simplified (**Supplementary Figure 5A**). In terms of domain architecture, all members of the OLRR-XII family are characterized by the presence of the PLN00113 superfamily domain. Beyond this core conserved domain, domain composition exhibits lineage-specific diversification: a subset of LRR-XII genes additionally, harbor accessory domains, including the PKc_like superfamily and SPS1 superfamily, which may contribute to functional specialization within the family (**Supplementary Figure 5B**).

### 3.5 The ODUF26 family

**Figure 3:**
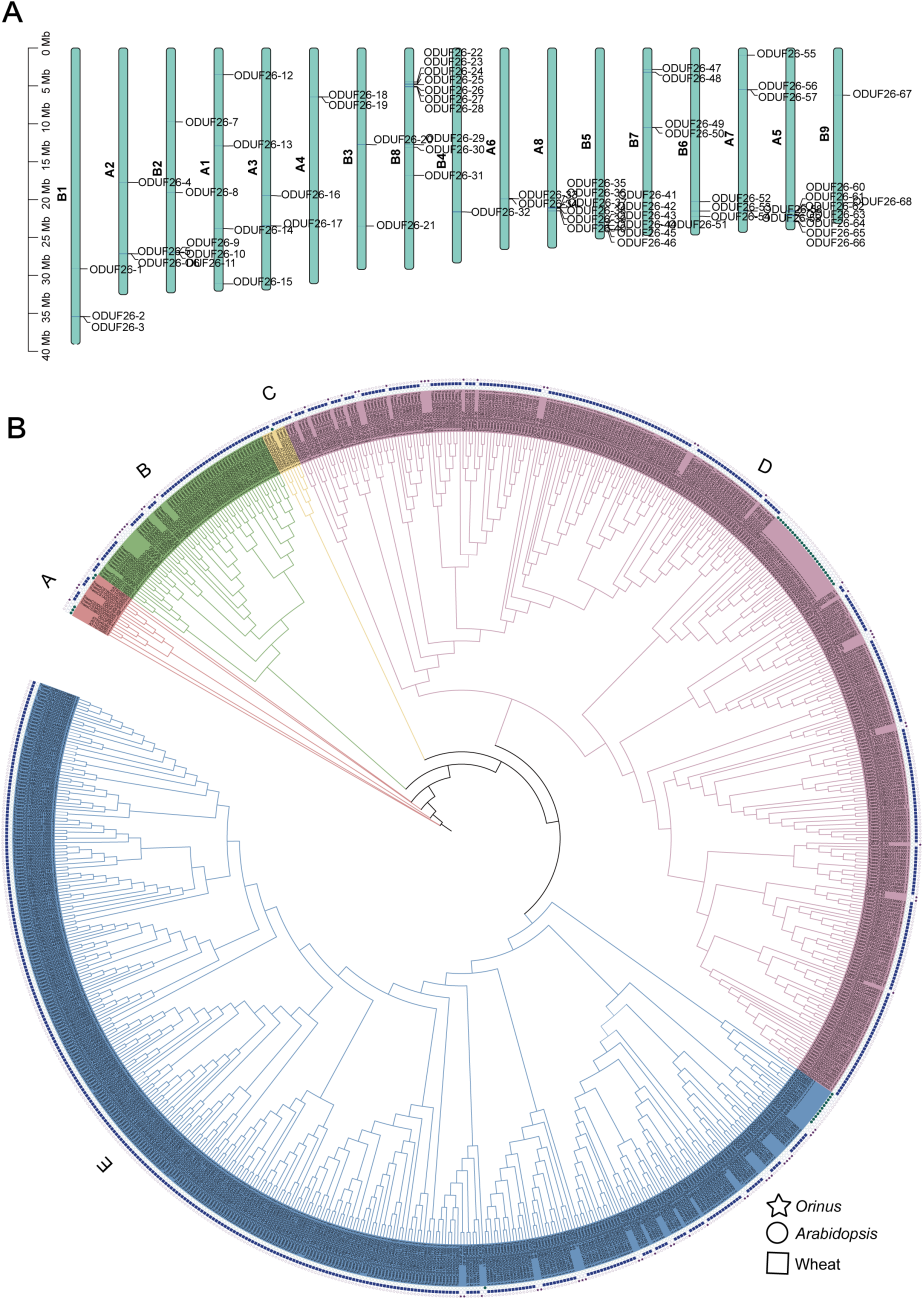
Evolutionary analysis of the DUF26 family. **(A)** Chromosomal distribution of ODUF26 genes. **(B)** Phylogenetic tree of DUF26 in *Orinus*, *Arabidopsis* and wheat.

The DUF26 gene family of *Orinus* receptor-like kinases (ODUF26) comprises a total of 69 genes, and 68 are mapped onto chromosomes and named DUF26–1 to DUF26-68. The ODUF26 gene family members were unevenly distributed across 17 chromosomes of *Orinus*. Chromosome B8 showed the highest gene density, harboring 10 ODUF26 genes. Chromosome A8 and A5 also contained a concentrated cluster, while chromosomes like A2, B1, an A3 and B3 carried relatively few genes. Particularly, tandem duplication events were prominent that multiple adjacent gene clusters (ODUF26-22/-23/-24/-25/-26/-27/-28 on B8, ODUF26-35/-36/-37/-38/-39/-40 on A8) were observed, indicating that tandem duplication contributed to the expansion of the ODUF26 gene family in *Orinus* (**Figure 3A**). The phylogenetic tree of the DUF26 gene family from *Orinus*, wheat, and *Arabidopsis* resolved the *Orinus* DUF26 genes into A to E five distinct clades, designated as Groups A to E. DUF26 genes from all three species are represented in Groups A, D and E, while Group B is specific to wheat and *Orinus*, and Group C to wheat and *Arabidopsis*. This intricate distribution pattern directly reflects the evolutionary diversity of the ODUF26 gene family across different species (**Figure 3B**).

Conserved motif analysis of the ODUF26 family, signature motifs such as Motif 3, Motif 4 and Motif 7 were widely distributed in most members and typically arranged in fixed sequential combinations (**Supplementary Figure 6**). Domain prediction, as illustrated in **Supplementary Figure 7**, showed that the ODUF26 family harbors a conserved STKc_IRAK domain and a Gnk2-like domain, along with a variety of accessory domains.

### 3.6 The OWAK family

**Figure 4:**
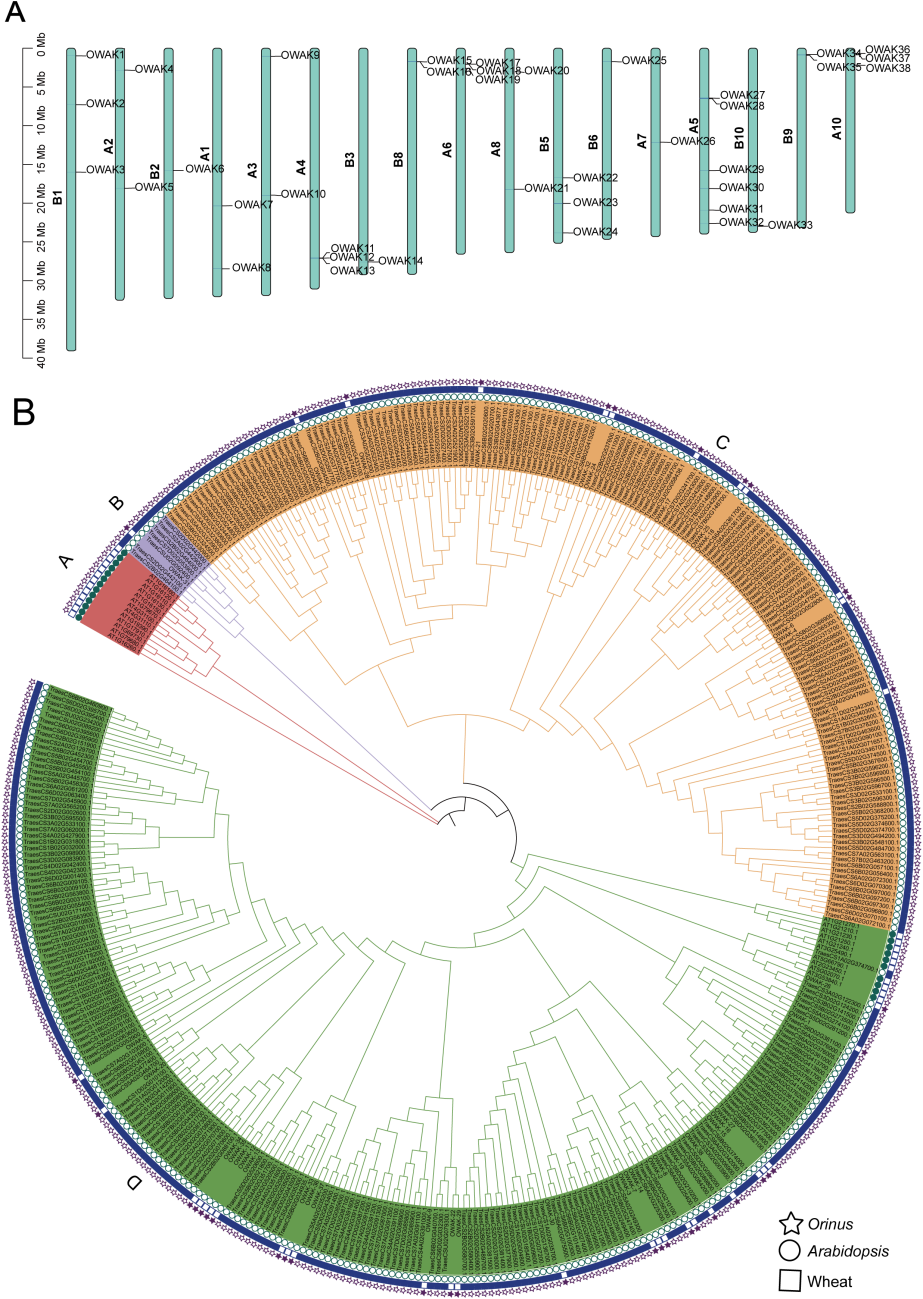
Evolutionary analysis of the LRR-III family. **(A)** Chromosomal distribution of OWAK genes. **(B)** Phylogenetic tree of WAK in *Orinus*, *Arabidopsis* and wheat.

Chromosomal mapping of the 38 *Orinus* WAK (OWAK) genes revealed their non-uniform distribution across 17 chromosomes, ranged from 1 to 6 genes on most to pronounced peaks on A6 and A10. Consistent with the distribution pattern, tandem duplications were identified in the OWAK gene family. Tandem repeat gene pairs were found on chromosomes A5, A6, A10, B8 and B9. Especially, a three-gene cluster was detected on A4 (**Figure 4A**). These clustered arrangements, combined with the scattered distribution of single OWAK genes on chromosomes, suggest that both tandem duplication and discrete gene retention contributed to the current genomic architecture of the OWAK family. Similarly, we conducted a systematic phylogenetic analysis of the identified OWAK genes using three species sequences. The WAK proteins of these three species can be classified into 4 clusters (A-D) based on the conservation of the conservation domain. Compared to wheat, *Orinus* and *Arabidopsis* has relatively few WAK proteins and showed a specific distribution that only exist in group A and D (**Figure 4B**). This implies that OWAK members undergo differentiation between monocotyledons and dicotyledons and may possess distinct functions in speciation adaptation.

Motif and domain analysis (**Supplementary Figure 8**) indicated that the main conserved motifs of the OWAK family are Motif 1, 5, and 10, and its family-specific conserved domain is GUB_WAK_bind; the conserved motifs and domains of OWAK proteins show similar consistency in structural distribution, suggesting functional uniformity among family members.

### 3.7 Conserved protein motif and domain analysis of the OLRR-III gene family

**Figure 5:**
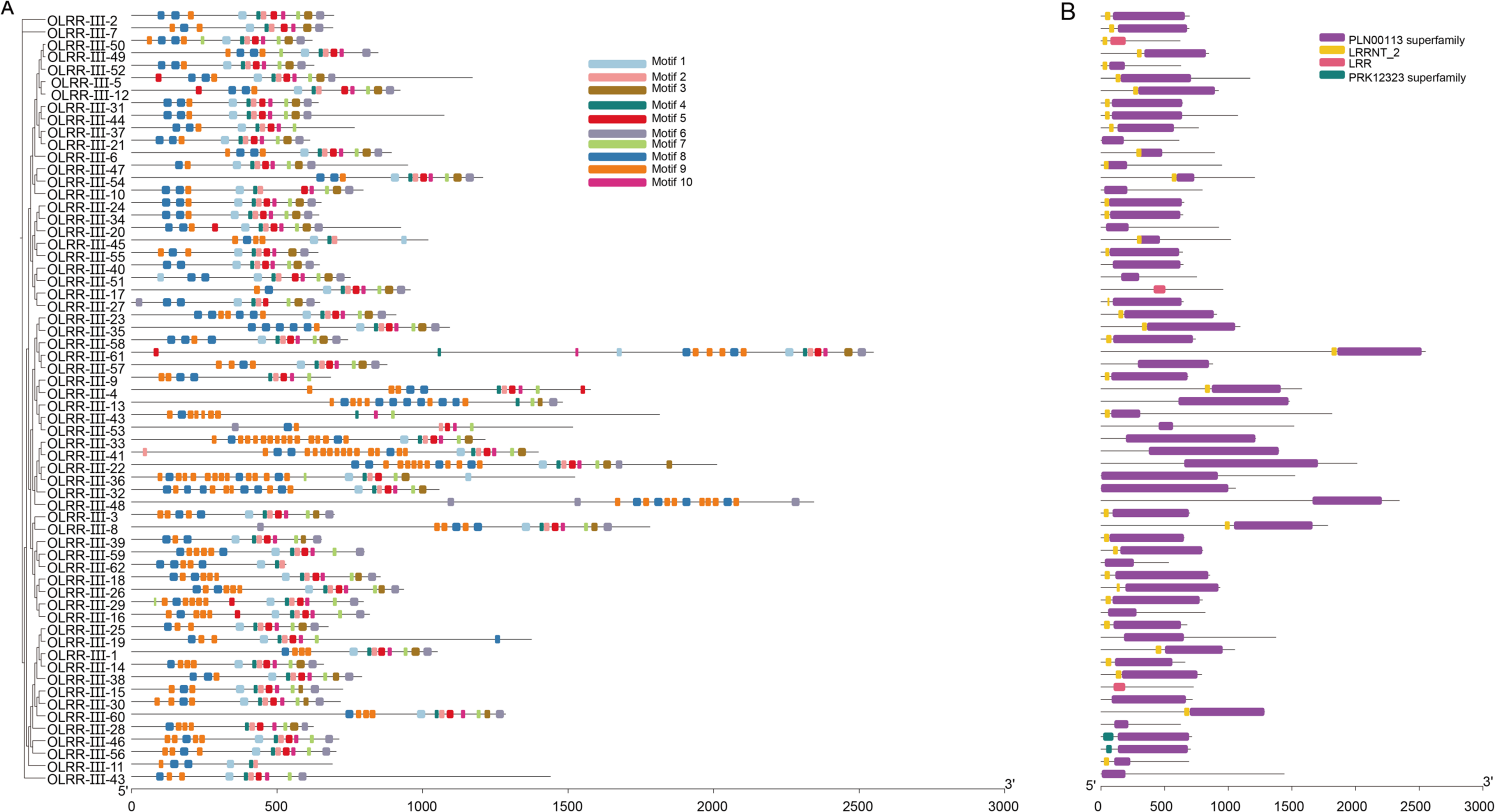
The motifs and domains analysis of OLRR-III. **(A)** OLRR-III gene domains and **(B)** motifs. Different motifs are indicated by different colors and numbers.

To clarify the functional characteristics of the OLRR-III genes, we conducted a combined analysis of their conserved motifs and protein domains. Protein motifs were highly conserved amino acid residues and considered to have functional and structural roles in active proteins (Bao et al., 2025). Ten conserved motifs were identified in OLRR-III subfamily and these motifs displayed highly similar distribution patterns across the protein sequences (**Figure 5A**). We also performed a conserved domain analysis on the 89 identified OLRR-III genes, and 62 members contained complete LRR domains (**Figure 5B**). The presence and architecture of these domains further support their role as functional receptor kinases. Together, the conservation of both motifs and domains indicates that the OLRR-III genes have maintained core functional units throughout evolution. This conservation likely underpins their critical and non-redundant roles in the biological processes of *Orinus*.

### 3.8 Synteny analysis of five ORLKs subfamilies among *Orinus*, wheat and *Arabidopsis*

**Figure 6:**
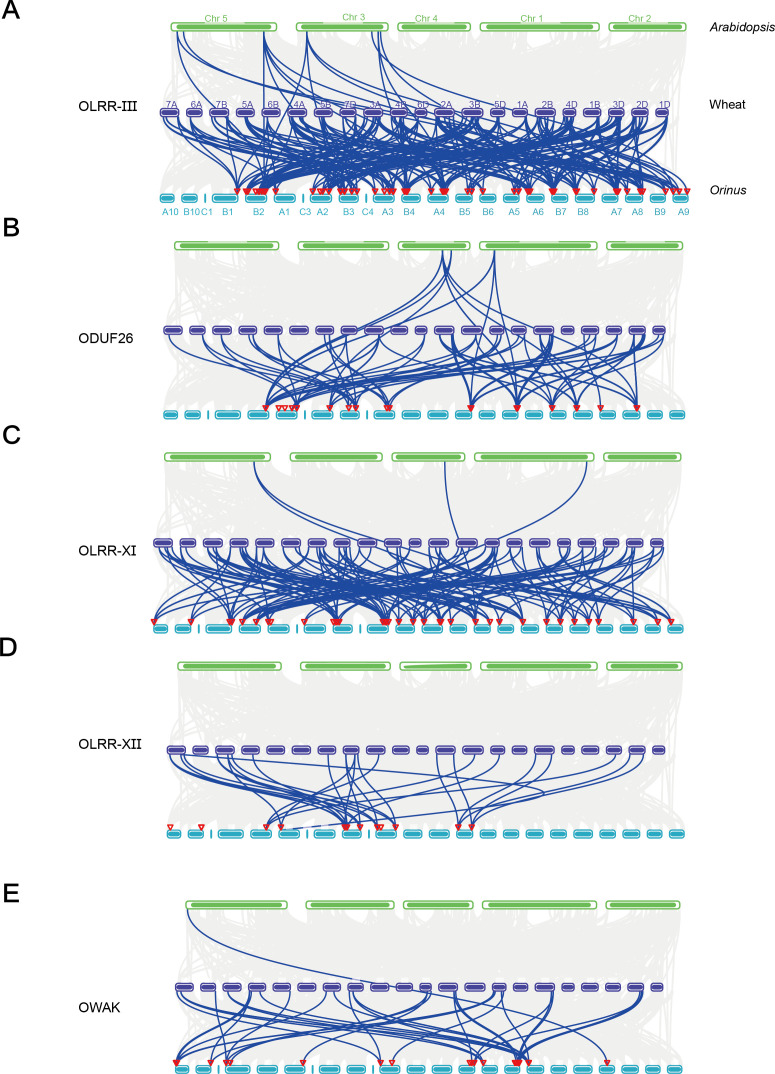
Interspecific collinearity analysis of ORLKs. **(A)** Collinearity of the OLRR-III family in wheat and *Arabidopsis*. **(B)** Collinearity of the ODUF26 family in wheat and *Arabidopsis*. **(C)** Collinearity of the OLRR-XI family in wheat and *Arabidopsis*. **(D)** Collinearity of the OLRR-III family in wheat and *Arabidopsis*. **(E)** Collinearity of the OWAK family in wheat and *Arabidopsis*. To avoid text clutter in the figure, chromosome numbers and species names should only appear once.

Gene duplication is a major driver of plant evolution (Vanneste et al., 2014). To explore the collinearity relationships in five ORLK subfamilies among *Orinus*, wheat and *Arabidopsis*, we employed MCScanX in TBtools. However, the distribution patterns of collinear gene pairs differed notably among subfamilies. The OLRR-III family showed dense collinear connections in wheat (**Figure 6A**), while identifying 15 homologous gene pairs in *Arabidopsis.* ODUF26 exhibited a more scattered pattern in wheat, but 9 pairs in *Arabidopsis* (**Figure 6B**). The OLRR-XI family contained a relatively high number of collinear gene pairs in wheat, just 4 pairs in *Arabidopsis* (**Figure 6C**), while OLRR-XII, with fewer members were displayed in wheat and none in *Arabidopsis* (**Figure 6D**). The OWAK family exhibited a notably dense collinearity pattern in wheat, however, only 1 in *Arabidopsis* (**Figure 6E**). These results highlight stronger genomic conservation between *Orinus* and wheat, both monocots, than with the dicot *Arabidopsis*, reflecting divergence after the monocot-dicot split.

### 3.9 Ka/Ks analysis of ORLKs

To investigate the evolutionary selection pressure acting on ORLK genes, we calculated Ka/Ks ratios for 1:1 orthologous ORLK pairs between *Orinus* and wheat. The Ka/Ks distribution revealed that nearly all ORLK orthologs were under strong purifying selection, with only a small subset showing signs of positive selection (**Supplementary Figure 9**). These findings suggest that RLK genes are highly conserved during evolution, while a limited number of ORLKs may have undergone adaptive evolution to facilitate high-altitude adaptation in *Orinus.*

### 3.10 Expression analysis of differential genes of ORLK in two species of *Orinus*

**Figure 7:**
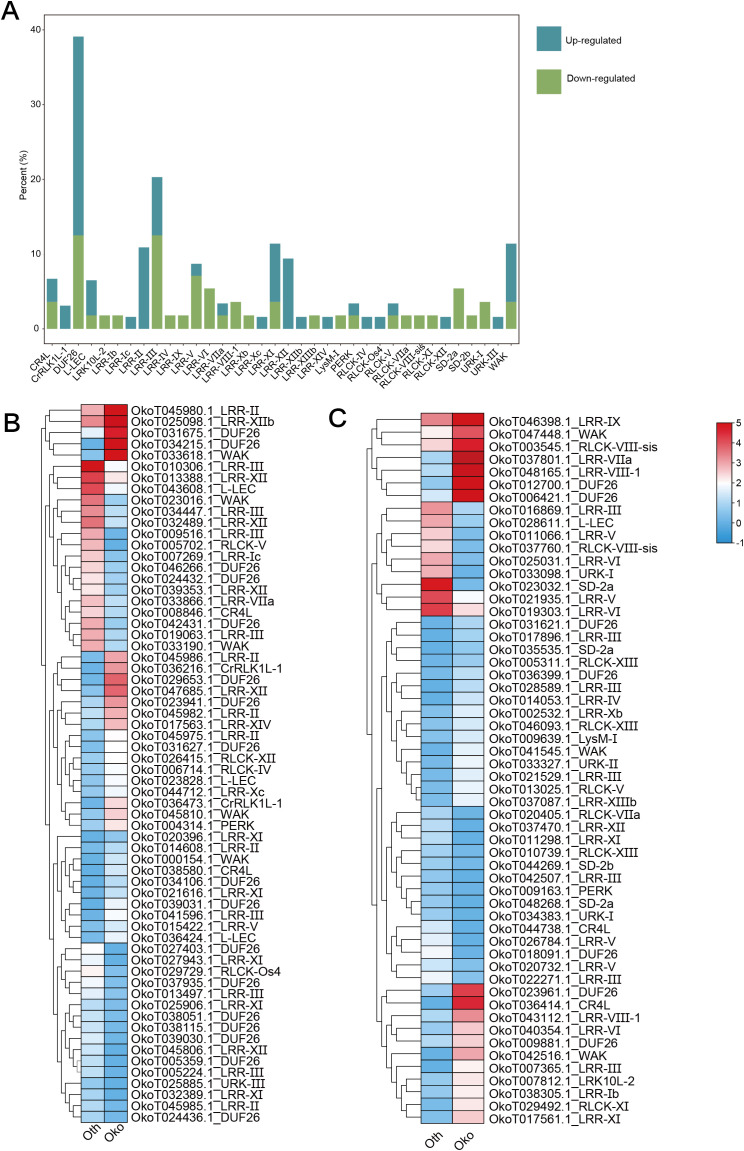
Expression profile of DEGs. **(A)** Stacked bar chart of DEGs by percentage of quantity. **(B)** Heatmap of up-regulated DEGs. **(C)** Heatmap of down-regulated DEGs. The heatmaps show the expression patterns of RLK genes across samples. Raw expression counts were subjected to log_2_ transformation and then row-wise Z-score normalization to standardize gene expression levels. The color scale (blue to red) represents the normalized expression values, with blue indicating low expression and red indicating high expression.

Focusing on the potential biological roles of RLKs in *Orinus*, the expression patterns of all ORLK families in the two *Orinus* species were investigated using public RNA-Seq data. Among the 647 ORLK genes analyzed, 120 differentially expressed genes (DEGs) emerged (**Supplementary Table 4**). And these DEGs belong to 36 ORLK subfamilies. Within the scope of the DEGs, 21 subfamilies of the ORLK family were up-regulated, the ODUF26 family had the highest proportion, while 28 subfamilies were down-regulated, ODUF26 and OLRR-III families had the same proportion (**Figure 7A**). Some ORLKs families such as OLRR-XII, OLRR-III, ODUF26 were more likely to appear frequently in RLKs that were expressed higher in *O. kokonoricus* (**Figure 7B**). ODUF26 and OWAK subfamilies more present in DEGs that were expressed higher in *O. thoroldii* (**Figure 7C**). Interestingly, CrRLK1L-1, LRR-XIIb and LRR-XIV only existed in up-regulated DEGs while LRK10L-2 and LRR-VIII-1 only presented in down-regulated. It implied that altitude-driven selection pressures have shaped the directional expression of specific ORLK subfamilies.

### 3.11 *Cis*-acting element prediction in promoter regions of DEGs

**Figure 8:**
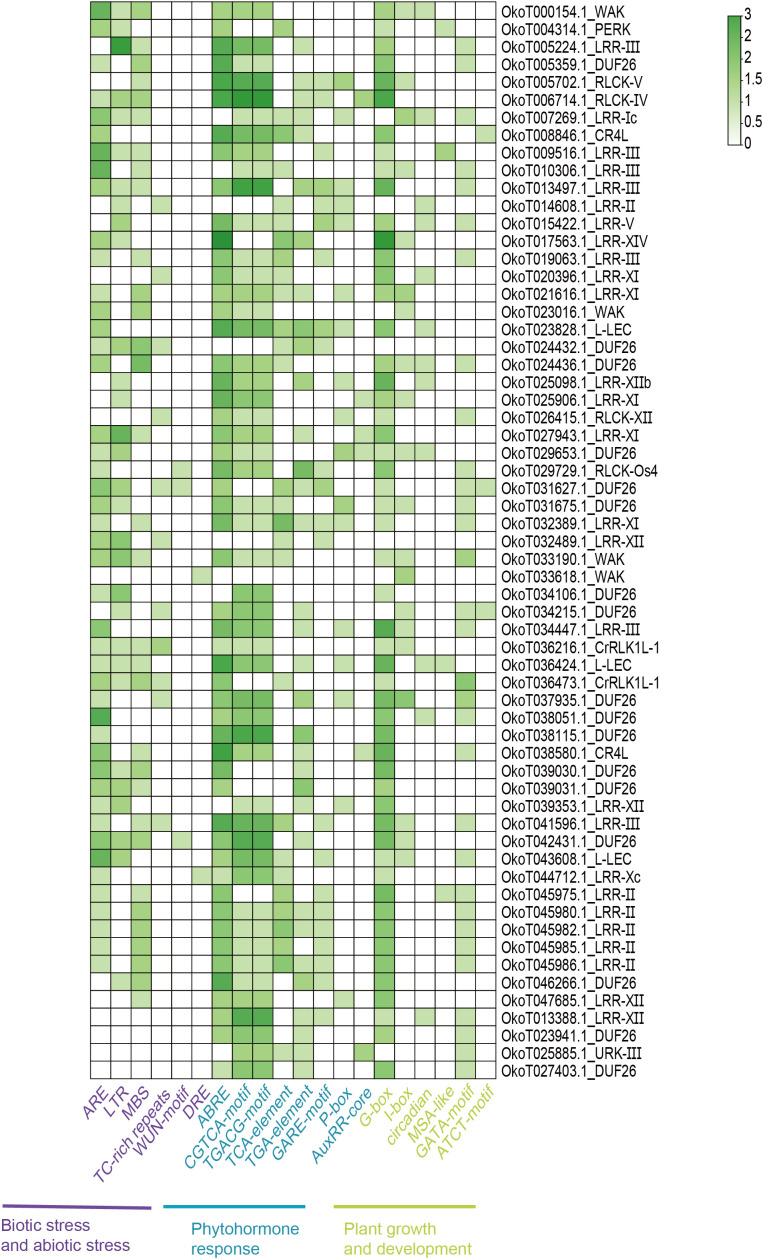
Distribution of cis-acting elements in the promoters of up-regulated s. The raw count numbers of cis-acting elements were first subjected to log_2_ transformation (base=2) to reduce data skewness, followed by row-wise Z-score normalization to standardize the relative abundance of each cis-element across gene groups.

**Figure 9:**
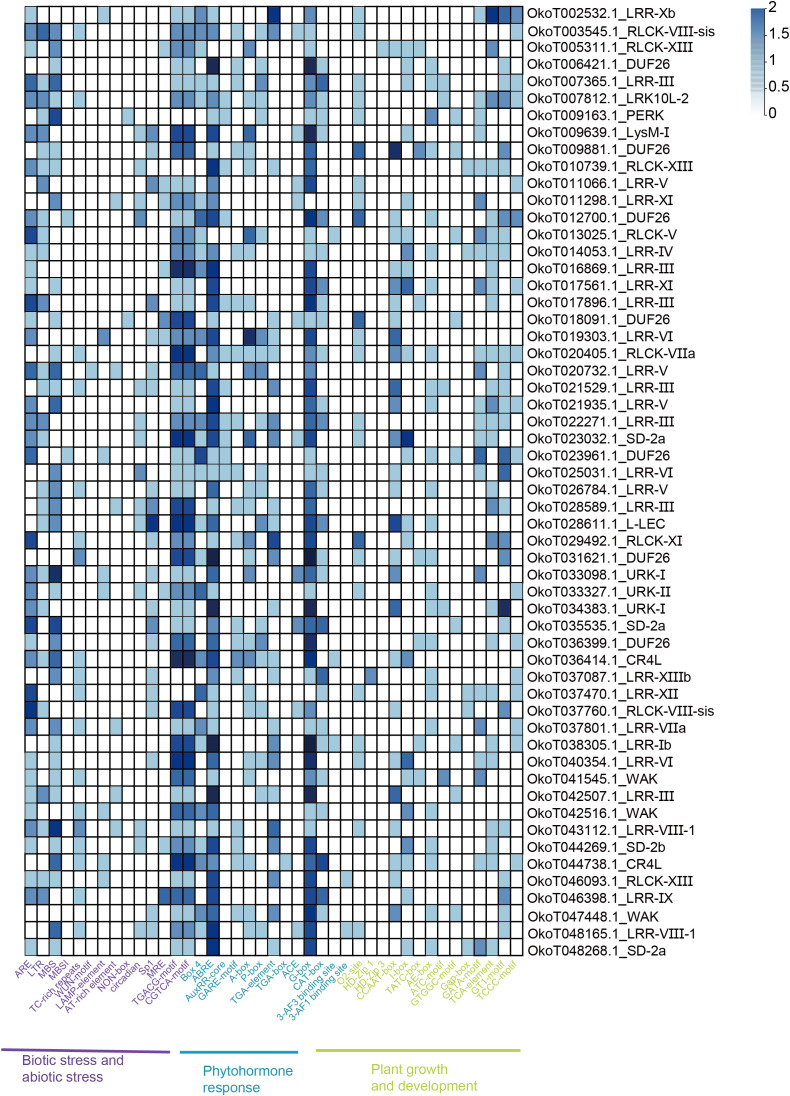
Distribution of cis-acting elements in the promoters of down-regulated DEGs. The raw count numbers of cis-acting elements were first subjected to log_2_ transformation (base=2) to reduce data skewness, followed by row-wise Z-score normalization to standardize the relative abundance of each cis-element across gene groups.

To further characterize DEGs, we analyzed the 2.0 kb upstream sequences of DEGs using the PlantCARE database to identify *cis*-regulatory elements. We classified the *cis*-acting elements predicted from the up-regulated DEGs into three categories: stress-responsive, phytohormone-responsive, and growth-related (**Figure 8**). The number of *cis*-regulatory elements was predominantly concentrated in phytohormone-responsive elements, such as ABRE, TCA-element, and GARE-motif. G-box and GATA-motif were the two most abundant *cis*-acting elements associated with plant growth and development. ARE, LTR and MBS were the most predominant *cis*-acting elements involved in stress responses. The five ORLK subfamilies of interest, the overall distribution of *cis*-acting element numbers in OLRR-III, OLRR-XI, OLRR-XII and OWAK was also concentrated in the several element types mentioned previously, except that a small number of genes in the ODUF26 subfamily lacked stress-responsive elements (OkoT023941.1_DUF26 and OkoT027403.1_DUF26). Similarly, we classified the *cis*-acting elements of the down-regulated DEGs into three groups: stress, hormonal, and growth (**Figure 9**). The main types of *cis*-acting elements in the down-regulated genes were similar to those in the up-regulated genes, but there were also a few distinct element types (Sp1, Box-4 and GT1-motif).

### 3.12 GO and KEGG analysis of DEGs in *Orinus*

To further explore the functional characteristics of DEGs in *Orinus*, we performed GO and KEGG enrichment analyses. GO enrichment analysis revealed that these DEGs were significantly enriched in the molecular function term “transmembrane signaling receptor activity”, which is the core functional feature of DEGs, confirming their central role as transmembrane receptors for extracellular signal perception and transduction. In the biological process category, “signal transduction”, “cellular response to stimulus”, “primary metabolic process” and “developmental growth” were prominently enriched (**Supplementary Figure 10A**). Consistent with the GO results, KEGG pathway enrichment analysis showed that the most significantly enriched pathway was “Protein kinases”, followed by “Signal transduction” and the broad category of “Environmental Information Processing” (**Supplementary Figure 10B**).

## 4 Discussion

RLKs constitute one of the largest gene families in plants and play pivotal roles in mediating signal transduction pathways involved in growth, development, and stress responses (Jones and Dangl, 2006); this family originated with the first discovery of a maize protein kinase analogous to animal receptor tyrosine kinases (Walker and Zhang, 1990). To clarify the evolutionary characteristics and adaptive significance of ORLKs, we identified 647 ORLKs in this study. Notably, this number accounts for merely one-fifth of the 3424 RLKs in hexaploid wheat and is far below the theoretical expectation for a tetraploid species, yet it is much closer to the 610 typical RLKs in *Arabidopsis* (Shiu and Bleecker, 2001). It is well established that polyploidization is often followed by genome shock and diploidization, which cause extensive gene loss (Feldman and Levy, 2012). Moreover, previous phylogenomic analyses have confirmed that RLK expansion originated in early plant evolution, laying the foundation for plants to adapt to diverse habitats (Gong and Han, 2021). Combining these findings with our identification results, we speculate that the reduced number of ORLKs has little to do with polyploidization, but is associated with its own environmental adaptation processes, which may have led to adaptive sequence divergence of RLKs during the evolution of *Orinus*.

Further classification showed that these 647 ORLKs can be divided into 52 subfamilies. This number is 6 fewer than the 58 subfamilies identified in wheat, and importantly, all 52 subfamilies are shared by wheat and *Orinus*. This indicates that *Orinus* has not evolved any entirely novel subfamilies beyond those present in wheat, suggesting that *de novo* subfamily differentiation of RLKs may have occurred in the wheat lineage. In contrast, the 52 ORLK subfamilies are 9 more than the 43 subfamilies in *Arabidopsis*, which implies that monocot plants (wheat and *Orinus*) may possess greater diversity in RLK subfamilies compared with dicot plants (*Arabidopsis*) (Fischer et al., 2016). This indicates that the RLK subfamilies shared with wheat may retain the basic stress response pathways of Poaceae plants, while the increased subfamily diversity compared with *Arabidopsis* is likely an adaptive expansion in response to high-altitude-specific stresses, which lays a molecular foundation for the unique high-altitude stress resistance of *Orinus.*

Phylogenetic analysis was conducted on five core ORLK subfamilies, namely OLRR-III, ODUF26, OLRR-XI, OLRR-XII, and OWAK, together with their homologous sequences from wheat and *Arabidopsis*, which revealed distinct evolutionary relationships among these subfamilies. All five gene families exhibit uneven chromosomal distribution in *Orinus*, rely primarily on tandem duplication as the major mechanism for family expansion, show high conservation of protein domains and motifs, and have undergone pronounced species-specific evolutionary divergence. Meanwhile, they differ significantly from each other in terms of gene number, expansion intensity, evolutionary gene loss, domain diversity, and the extent of their association with environmental adaptation. Collectively, these five subfamilies form the evolutionary and functional foundation of the RLK system in *Orinus*, confirming that lineage-specific functional diversification of RLKs is a key adaptive strategy for *Orinus* to cope with its environment. Importantly, OLRR-III and ODUF26 are *Orinus*’ largest ORLK subfamilies, implying their core roles in plateau environmental adaptation. This inference is supported by previous studies: in maize, the LRR-RLK gene ZmMIK2 is involved in mediating resistance to drought and salt stresses (Yang et al., 2025), while LRR-RLKs in wheat contribute to antiviral defense (Liu et al., 2022). The identification of tandem duplication clusters in these ORLK subfamilies further confirms the conserved role of duplication events in driving RLK family expansion (Lehti-Shiu et al., 2009). Persistent duplication events likely to have provided sufficient genetic material for the functional divergence of ORLKs, thereby enhancing the adaptive capacity of *Orinus* to extreme abiotic stresses in plateau environment. However, the specific functions of duplicated ORLK genes, their regulatory networks, and the molecular mechanisms underlying their functional divergence remain unclear, which necessitates further functional verification experiments in future studies. To further elucidate the evolutionary selection regimes shaping ORLK genes, we calculated the Ka and Ks substitution rates for one-to-one orthologous pairs between *Orinus* and wheat. Most ORLK orthologs exhibited Ka/Ks < 1, indicating strong purifying selection and high functional conservation during evolution. Meanwhile, a small subset of ORLKs showed Ka/Ks > 1, suggesting that these genes underwent positive selection, which likely contributed to adaptive evolution in response to high-altitude stresses.

Synteny analysis revealed extensive collinearity between *Orinus* and wheat ORLK subfamilies, but little with *Arabidopsis*. This reflects functional divergence, attributed to monocot-dicot differences (Mangelsen et al., 2008) and genome complexity—wheat’s allohexaploid genome versus *Arabidopsis*’ small, simple genome (Brenchley et al., 2012). The five subfamilies retain greater conservation in Poaceae, diverging significantly post-monocot-dicot split. Their specialized functions are inferred: OLRR mediates cold stress signaling; ODUF26, also called Cysteine-rich Receptor-like Kinases (CRKs) regulates transcription via hormone and environmental signals, aiding drought and UV tolerance (Wrzaczek et al., 2010); OWAK synergize in abiotic responses and cell wall perception, forming a plateau adaptation signal network. Differential expression analysis identified 120 DEGs between *O. thoroldii* and *O. kokonoricus* (64 up, 56 down in *O. kokonoricus*). DEGs distribution reflect ORLKs’ role in adaptive differentiation, driven by habitat differences—*O. kokonoricus* grows at lower altitudes, with differential expression adapting to harsh conditions. Up-regulated DEGs concentrate in OLRR-XII, OLRR-III, ODUF26, and OL-LEC; down-regulated in ODUF26 and OWAK. AhLRR-RLK265 of peanut (*Arachis hypogaea* L.) enhances salt and drought tolerance (Wang et al., 2024), and DUF26 (CRK) of *Arabidopsis* protects against apoplastic oxidative stress (Idänheimo et al., 2014). Thus, OLRR-III and ODUF26 up-regulation boosts abiotic stress transduction, while ODUF26 and OWAK down-regulation reduces redundancy for precise stress network regulation and synergistically forming *O. kokonoricus*’ adaptation basis.

To gain a comprehensive overview of the biological functions of DEGs, we performed GO and KEGG enrichment analyses. GO terms including “transmembrane signaling receptor activity”, “signal transduction”, “cellular response to stimulus”, and “primary metabolic process” were significantly enriched. Correspondingly, KEGG pathways related to “protein kinases”, “signal transduction”, and “environmental information processing” were highly over-represented. These results collectively verify that ORLKs function mainly as transmembrane receptors involved in signal perception, stress response, and environmental adaptation, consistent with their evolutionary characteristics revealed by Ka/Ks analysis.

*Cis*-regulatory element analysis revealed complex ORLK regulatory networks, with elements mediating environmental, hormonal, and transcriptional regulation (Sun et al., 2018). The promoter regions of DEGs contain elements associated with plant growth, hormonal regulation, and stress responses. Up-regulated promoters enrich gibberellin-responsive (P-box), ABA-responsive (ABRE, MBSI), stress-responsive (ARE, MBS, DRE, LTR), and light/developmental elements. ABA elements aid drought adaptation (Zha et al., 2025), enabling ORLKs to integrate signals for spatiotemporal expression and stress-hormone crosstalk. Down-regulated promoters include low-temperature-responsive elements (LTR), binding cold-responsive transcription factors to initiate cascades (Dunn et al., 1998), forming *Orinus*’ low-temperature adaptation basis. Light-responsive SP1, though scarce, is indispensable—regulating plant lifecycle via photoreceptors (Gudi et al., 2023). HD-Zip elements are rare in down-regulated DEGs not like CGTCA-motif and G-box are high enriched in up-regulated, regulating leaf development (Tu et al., 2022) and drought responses via ABA signaling (Ahangarani Farahani et al., 2025), reflecting a growth-stress trade-off strategy. Overall, ORLK differential expression between the two *Orinus* species results from *cis*-element-mediated synergistic regulation of multiple pathways. Environmental, hormonal, and transcriptional signals form a complex ORLK regulatory network, with DEG patterns determining adaptive differentiation to altitudinal habitats. This provides a typical case for studying non-model extremophyte environmental adaptation mechanisms.

## 5 Conclusions

In this study, we identified 647 ORLK genes (ORLKs) in the *Orinus* genome and the ORLKs were classified into 52 subfamilies. Furthermore, Ka/Ks-based evolutionary selection pressure analysis revealed that most ORLK orthologs were under strong purifying selection, with a small subset of genes showing signs of positive selection (Ka/Ks > 1), indicating adaptive evolution. We performed systematic analyses including phylogenetic reconstruction, conserved domain identification, and chromosomal localization. GO and KEGG pathway enrichment analyses showed that the differentially expressed ORLK genes were significantly enriched in transmembrane signaling receptor activity, and were mainly involved in environmental signal perception and transduction. Moreover, Ka/Ks ratio calculation indicated that the majority of ORLK genes were under purifying selection (Ka/Ks < 1), while a small number of genes exhibited positive selection signals (Ka/Ks > 1), which may be closely related to the adaptation of *Orinus* to extreme high-altitude environments. Collectively, these findings not only clarify the functional characteristics of ORLK genes but also provide a theoretical basis for exploring the molecular mechanisms of high-altitude adaptation in *Orinus*.

## Data Availability

Publicly available datasets were analyzed in this study. This data can be found here: The paired-end RNA-seq information for two species of Orinus, we used the NCBI Sequence Read Archive (Accession: PRJNA385721).

## Supplementary material

Supplementary Figure 1 Chromosomal Distribution and Species Phylogenetic Tree of OLRR-XI family.

Supplementary Figure 2 The conserved motifs of OLRR-XI family.

Supplementary Figure 3 The conserved domains of OLRR-XI family.

Supplementary Figure 4 Chromosomal Distribution and Species Phylogenetic Tree of OLRR-XII family.

Supplementary Figure 5 The conserved motifs and domains of the OLRR-XII family.

Supplementary Figure 6 The conserved motifs of ODUF26 family.

Supplementary Figure 7 The conserved domains of OLRR-XI family.

Supplementary Figure 8 The conserved motifs and domains of the OWAK family.

Supplementary Figure 9 The Ka/Ks analysis of ORLKs.

Supplementary Figure 10 The GO and KEGG enrichment analysis of DEGs in *Orinus*.

Supplementary Table 1 Detailed information of 647 predicted ORLKs.

Supplementary Table 2 Physicochemical property information of 647 ORLKs.

Supplementary Table 3 Prediction results of subcellular localization for 647 ORLKs.

Supplementary Table 4 Differentially expressed RLK genes and their FPKM values.

Supplementary Table 5 ORLK genomic sequences.

Supplementary Table 6 ORLK CDS sequences.

Supplementary Table 7 ORLK Protein sequences.
