# Supplementary material for: Deciphering the role of receptor-like kinases in the adaptation of Orinus species to the habitat of the Qinghai-Xizang (Tibet) Plateau
Source: Front Plant Sci. 2026 Apr 29;17:1810781. doi: 10.3389/fpls.2026.1810781 (PMC13168086; doi:10.3389/fpls.2026.1810781)
Supplement: Supplementary Figure 1 — Chromosomal Distribution and Species Phylogenetic Tree of OLRR-XI family. [file DataSheet1.pdf]

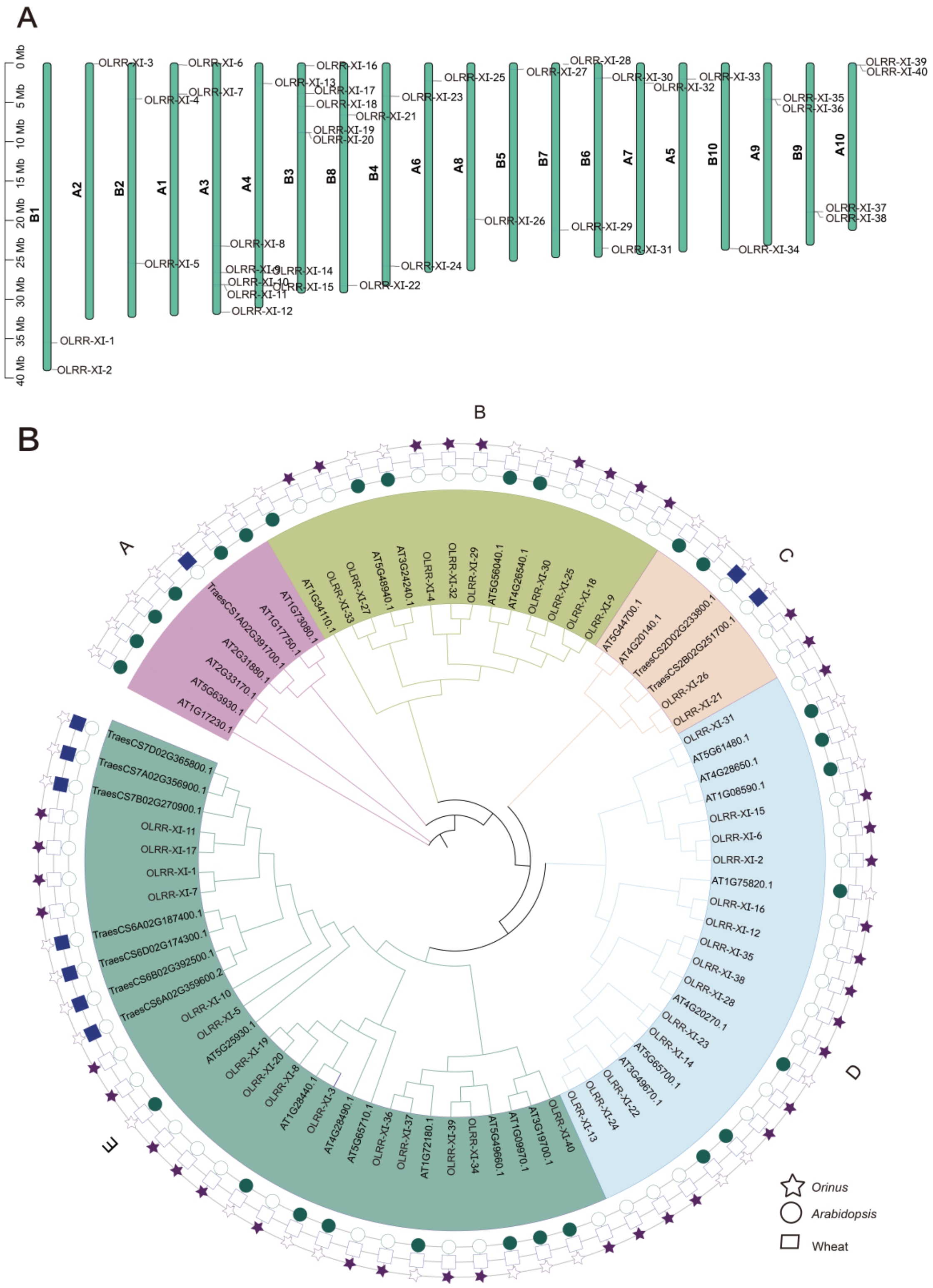

Supplementary Figure 1. Chromosomal localization and cross-species phylogenetic analysis of the OLRR-XI gene family**.** **(A)** Chromosomal localization of OLRR-XI genes. **(B)** Circular phylogenetic tree of LRR-XI proteins from *Orinus* (stars), *Arabidopsis* (circulars), and wheat (squares).

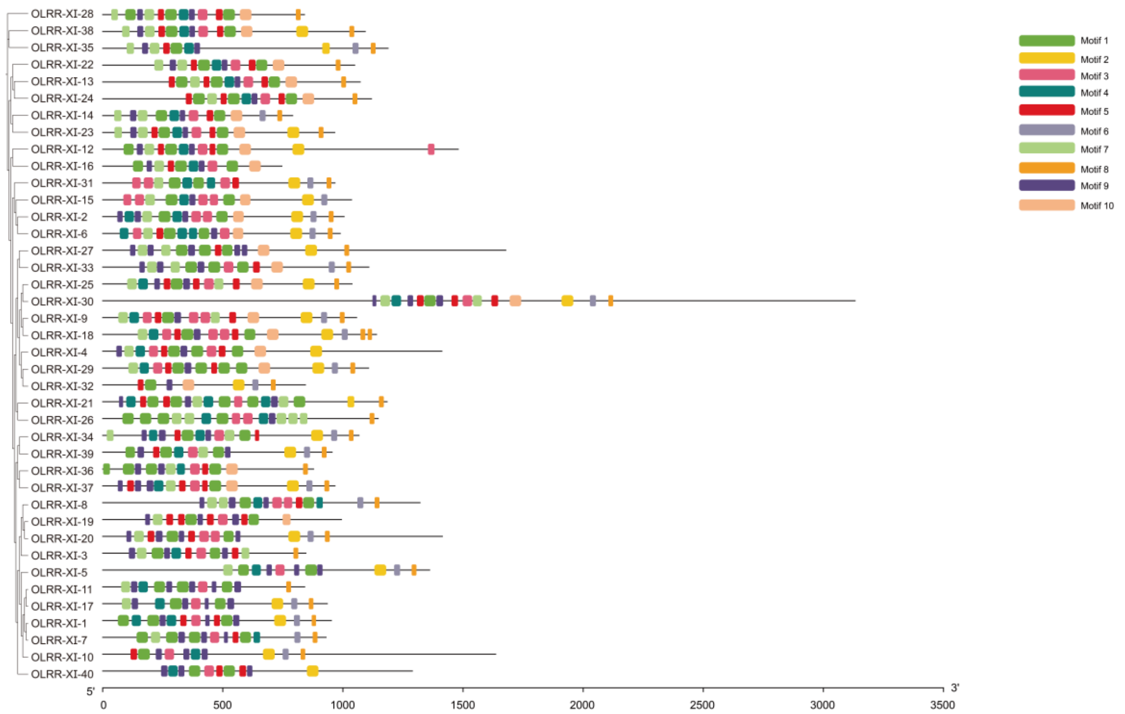

Supplementary Figure 2. Conserved protein motifs of OLRR-XI family.

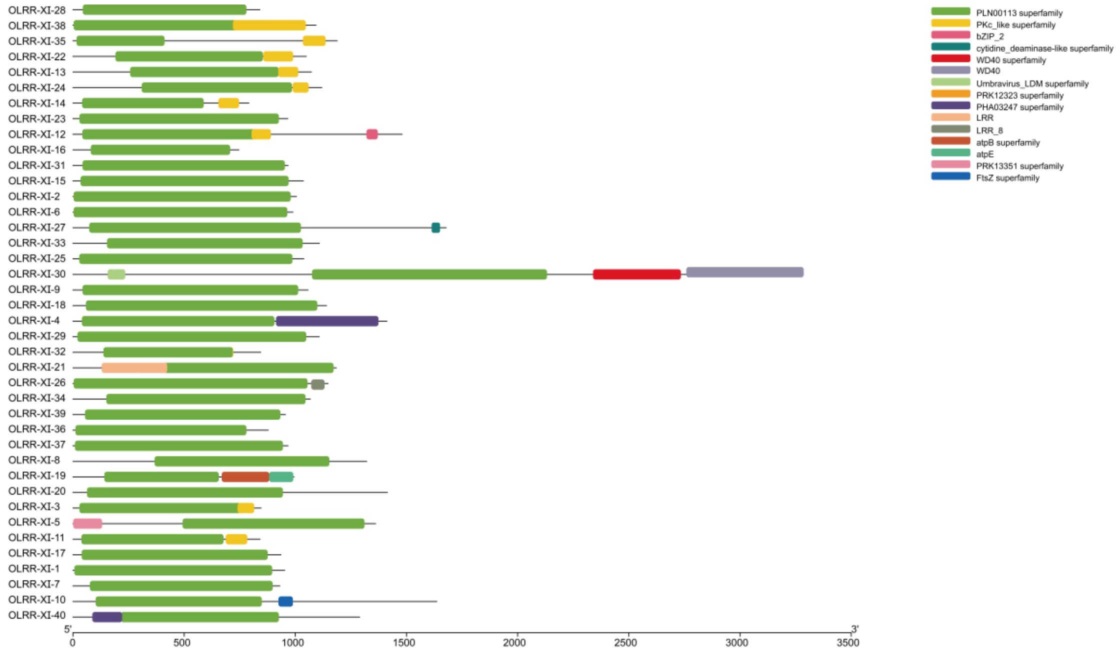

Supplementary Figure 3. Conserved domains of OLRR-XI family.

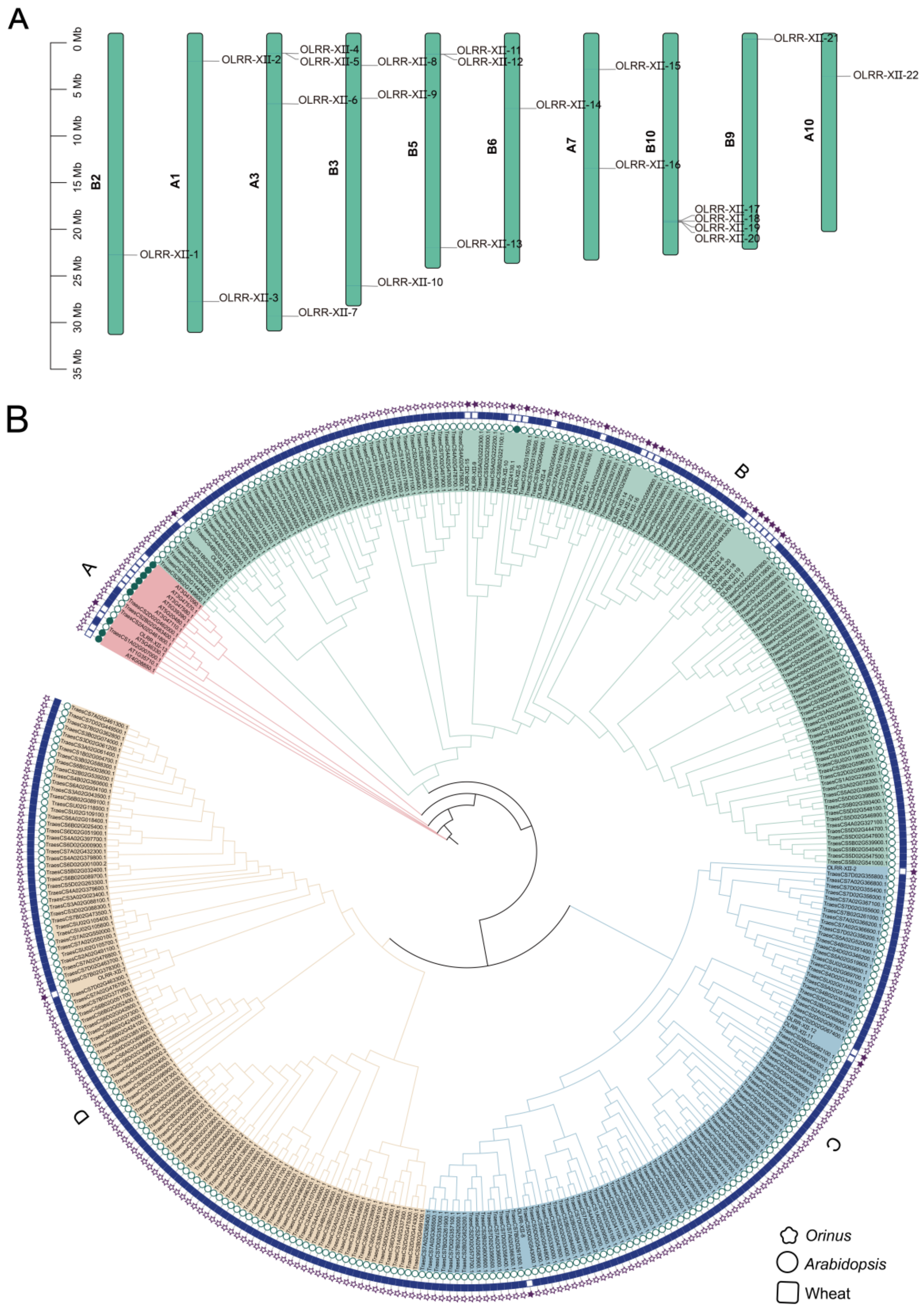

Supplementary Figure 4. Chromosomal localization and cross-species phylogenetic analysis of the OLRR-XII gene family. (A) Chromosomal localization of OLRR-XII genes. (B) Circular phylogenetic tree of LRR-XII proteins from *Orinus* (stars), *Arabidopsis* (circulars), and wheat (squares).

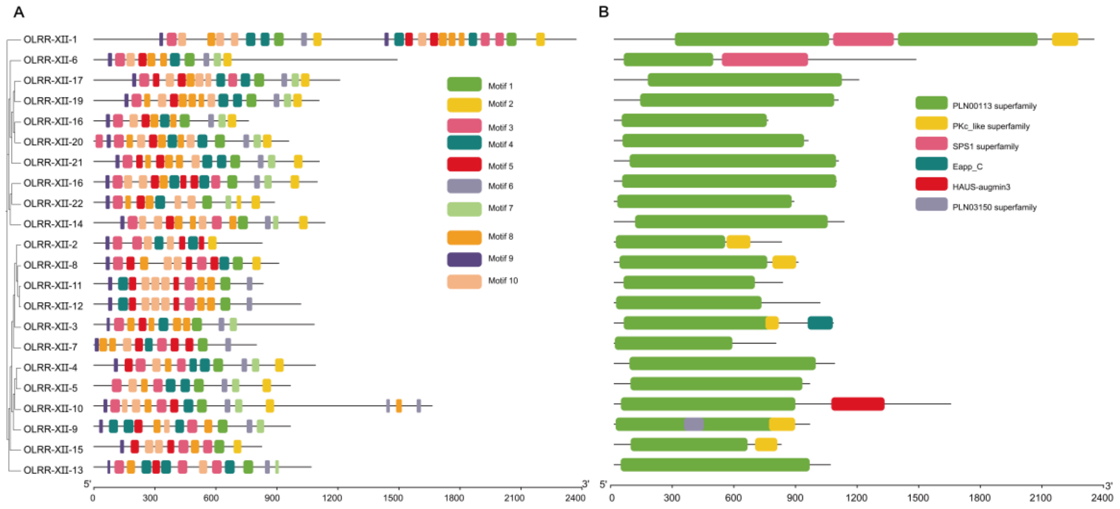

Supplementary Figure 5. (A) Conserved protein motifs and **(B)** domains of OLRR-XII family.

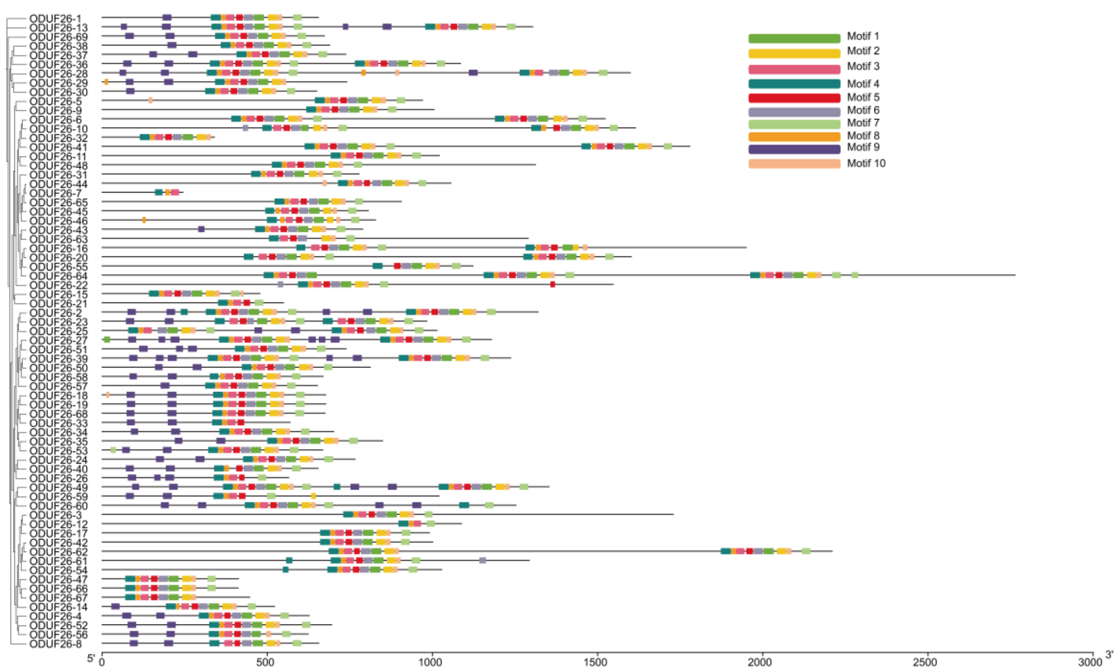

Supplementary Figure 6. Conserved protein motifs of ODUF26 family.

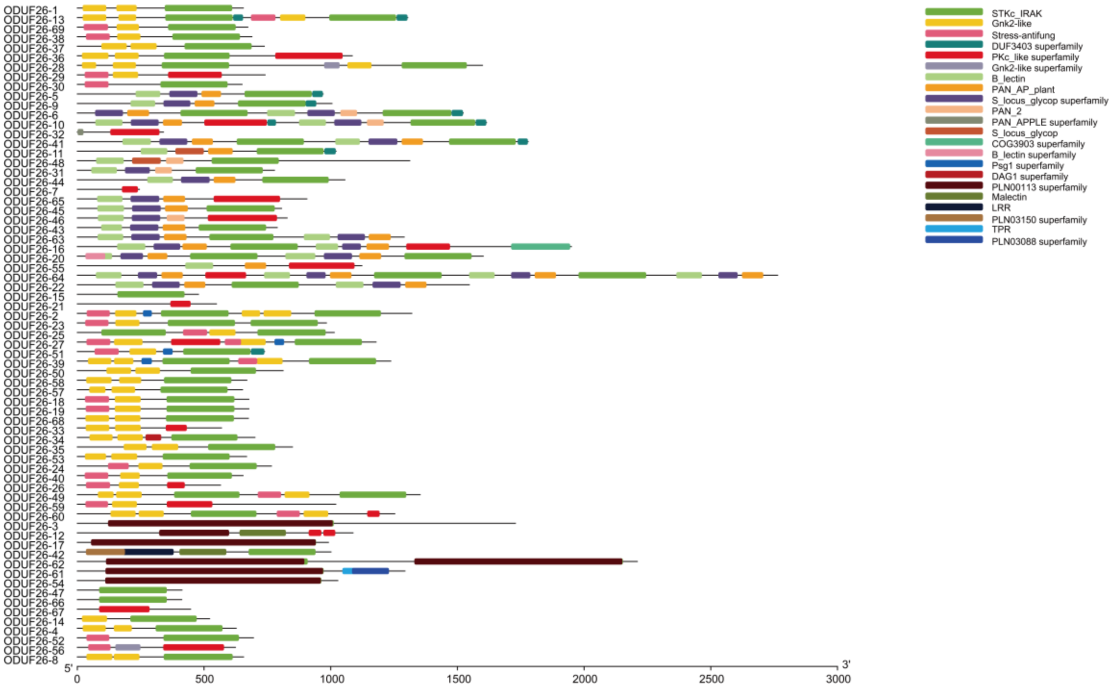

Supplementary Figure 7. Conserved domains of ODUF26 family.

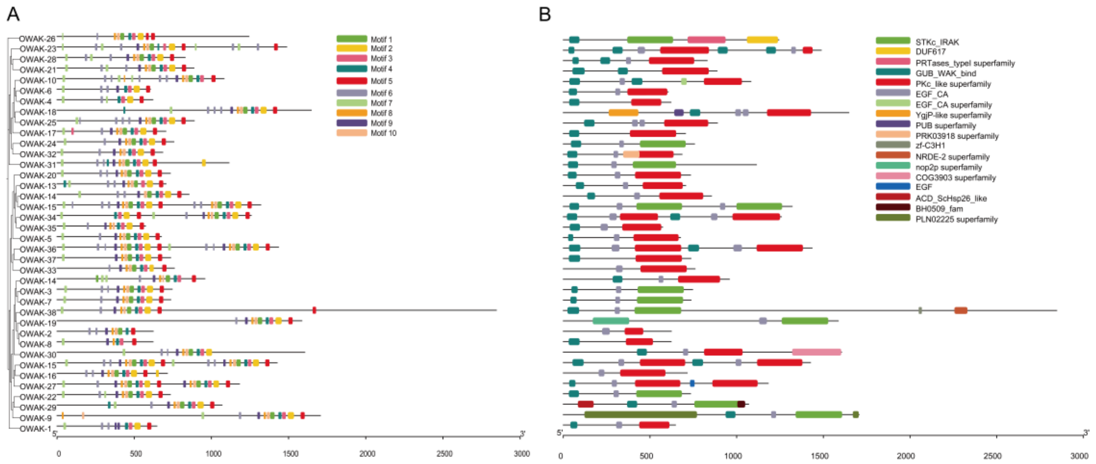

Supplementary Figure 8. (A) Conserved protein motifs and **(B)** domains of OWAK family.
