# Supplementary material for: Deciphering the role of receptor-like kinases in the adaptation of Orinus species to the habitat of the Qinghai-Xizang (Tibet) Plateau
Source: Front Plant Sci. 2026 Apr 29;17:1810781. doi: 10.3389/fpls.2026.1810781 (PMC13168086; doi:10.3389/fpls.2026.1810781)
Supplement: Supplementary Figure 9 — The Ka/Ks analysis of ORLKs. [file DataSheet4.pdf]

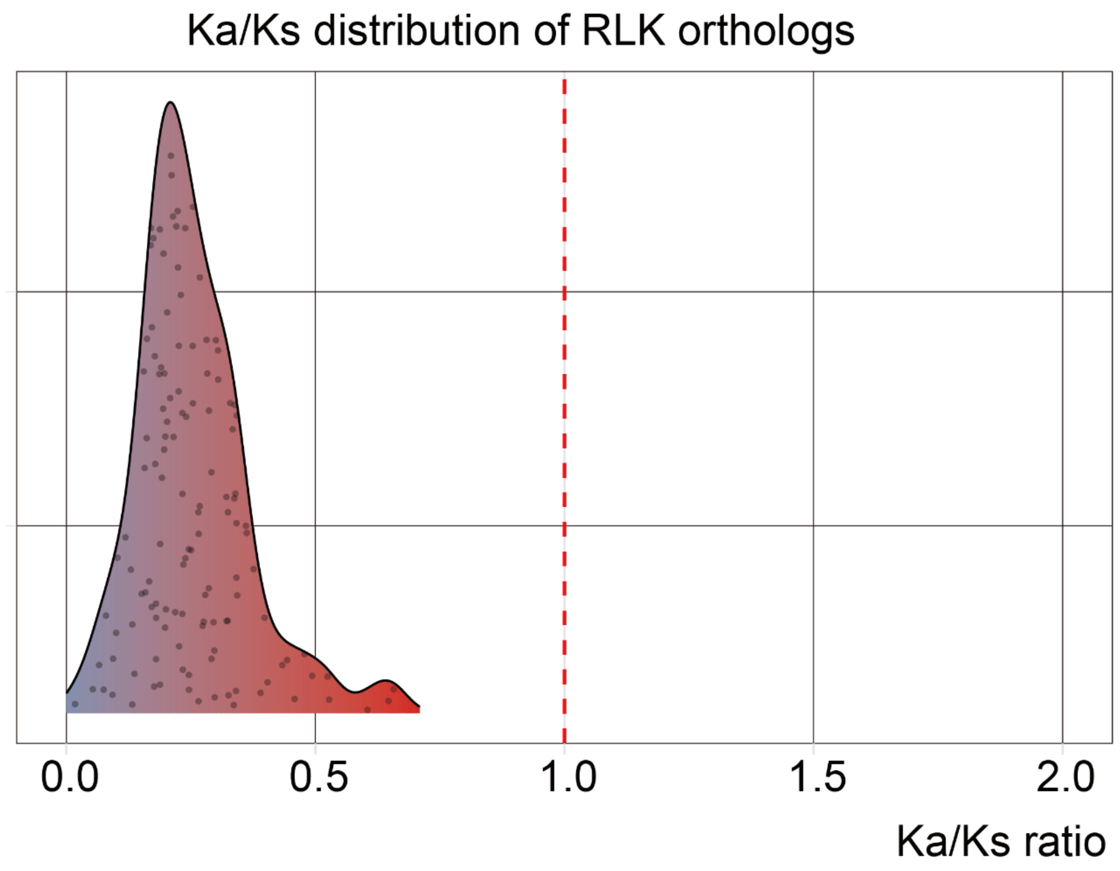

Supplementary Figure 9. Ka/Ks analysis of receptor-like kinases of *Orinus.*

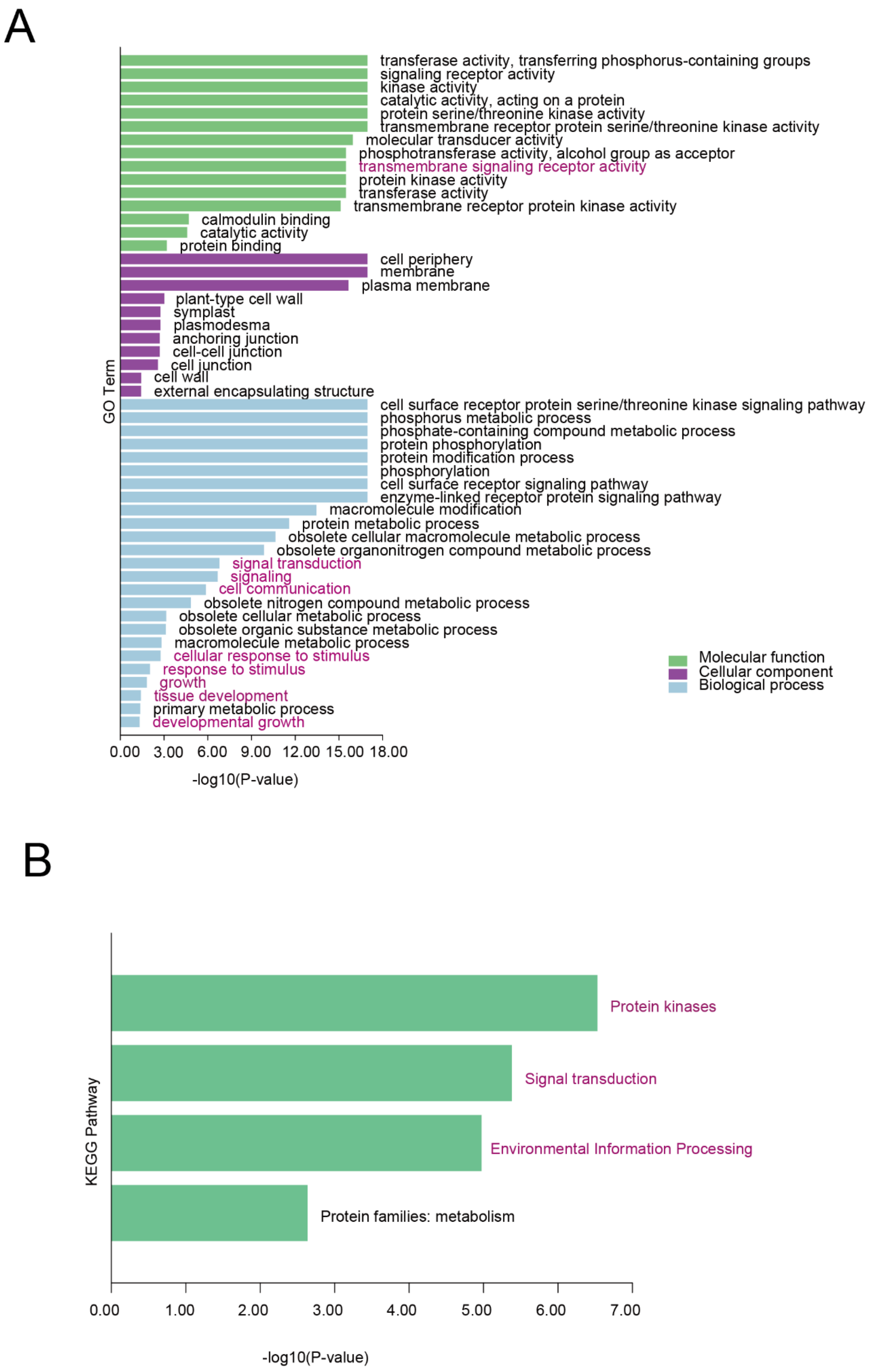

Supplementary Figure 10. Function and pathway enrichment analysis of DEGs.

(A) GO enrichment analysis of DEGs. (B) KEGG pathway enrichment analysis of DEGs.
